# Development and Characterization of a High Density SNP Genotyping Assay for Cattle

**DOI:** 10.1371/journal.pone.0005350

**Published:** 2009-04-24

**Authors:** Lakshmi K. Matukumalli, Cynthia T. Lawley, Robert D. Schnabel, Jeremy F. Taylor, Mark F. Allan, Michael P. Heaton, Jeff O'Connell, Stephen S. Moore, Timothy P. L. Smith, Tad S. Sonstegard, Curtis P. Van Tassell

**Affiliations:** 1 Department of Bioinformatics and Computational Biology, George Mason University, Manassas, Virginia, United States of America; 2 Bovine Functional Genomics Laboratory, United States Department of Agriculture, Agricultural Research Service, Beltsville, Maryland, United States of America; 3 Illumina, Inc., Hayward, California, United States of America; 4 Division of Animal Sciences, University of Missouri, Columbia, Missouri, United States of America; 5 U.S. Meat Animal Research Center, USDA, ARS, Clay Center, Nebraska, United States of America; 6 Department of Medicine, University of Maryland, Baltimore, Maryland, United States of America; 7 Department of Agricultural, Food and Nutritional Science, University of Alberta, Edmonton, Alberta, Canada; Ohio State University Medical Center, United States of America

## Abstract

The success of genome-wide association (GWA) studies for the detection of sequence variation affecting complex traits in human has spurred interest in the use of large-scale high-density single nucleotide polymorphism (SNP) genotyping for the identification of quantitative trait loci (QTL) and for marker-assisted selection in model and agricultural species. A cost-effective and efficient approach for the development of a custom genotyping assay interrogating 54,001 SNP loci to support GWA applications in cattle is described. A novel algorithm for achieving a compressed inter-marker interval distribution proved remarkably successful, with median interval of 37 kb and maximum predicted gap of <350 kb. The assay was tested on a panel of 576 animals from 21 cattle breeds and six outgroup species and revealed that from 39,765 to 46,492 SNP are polymorphic within individual breeds (average minor allele frequency (MAF) ranging from 0.24 to 0.27). The assay also identified 79 putative copy number variants in cattle. Utility for GWA was demonstrated by localizing known variation for coat color and the presence/absence of horns to their correct genomic locations. The combination of SNP selection and the novel spacing algorithm allows an efficient approach for the development of high-density genotyping platforms in species having full or even moderate quality draft sequence. Aspects of the approach can be exploited in species which lack an available genome sequence. The BovineSNP50 assay described here is commercially available from Illumina and provides a robust platform for mapping disease genes and QTL in cattle.

## Introduction

Cattle play a unique role in biology, serving as models for basic studies of metabolism, reproduction, and disease, as well as providing critical sources of human dietary protein and economic security (Bovine Genome Sequencing Initiative http://www.genome.gov/Pages/Research/Sequencing/SeqProposals/BovineSEQ.pdf). Moreover, large phenotypic datasets are routinely collected to assist beef and dairy production management and animal breeding decisions. These data represent a vast and relatively untapped resource to assist the investigation of the complex interplay of genetics and the environment, and the consequences of long-term selection for specific traits. Cattle have been a valuable model for studying mammalian physiology and comparative genomics, and represent the most thoroughly studied ruminant. Recently, a complete draft genome sequence, based on the DNA of a partially inbred individual from the Hereford breed and her sire, has been published (ftp://ftp.hgsc.bcm.tmc.edu/pub/data/Btaurus/). This draft sequence is an important tool for comparative and evolutionary studies. However, the full potential of genomic research in cattle cannot be realized without the ability to efficiently scan genome-wide for variation affecting the wide range of phenotypes recorded for this species.

Multiplex SNP genotyping allows the simultaneous high-throughput interrogation of hundreds of thousands of loci with high measurement precision at a cost that enables large-scale studies. SNP genotyping technology has created a surge in the number of genome-wide association (GWA) studies performed in human and has resulted in the identification of disease risk genes for complex polygenic traits [1]–[3]. High-density SNP genotyping or genome sequencing is available as a biomedical diagnostic for predicting individual predisposition to heritable genetic diseases, ushering in the era of “personal genomics”. Similar applications of these technologies in livestock species such as cattle will target the improvement of animal productivity, health and the accuracy of selection within genetic improvement programs using a modification of the GWA approach which has come to be called genome-wide selection (GWS; Meuwissen et al. 2001). The objective in using GWS is to use genomic data to supplement extensive sets of performance data to predict genetic merit values that are used for selection decisions by producers.

In cattle, GWA can be used to localize the genomic regions that contribute to natural genetic variation in any phenotypic trait. The identified target regions can then be fine-mapped at higher marker density to allow the efficient identification of candidate genes. The density of markers required for efficient GWA scans in a species is dependent on the average length of chromosome “blocks” possessing a high level of linkage disequilibrium (LD). The extent of LD between two loci is usually measured with statistics such as r^2^, which is the squared correlation between alleles present at two loci, typically on the same chromosome. Thus, what varies among species and certain populations within a species is the average length of genomic segments for which the r^2^ between terminal loci achieves a predetermined value, say r^2^ = 0.6. Within such blocks, one or a few markers (i.e., tagSNP) may act as proxies for predicting all, or most, of the haplotypes which are present within the entire block. It has been estimated that the average length of LD blocks is longer for cattle than for humans, due to significant population bottlenecks that occurred during domestication and the establishment of modern cattle breeds [4] and the recent exponential expansion of human populations [5]. While individual estimates vary, it appears that an average block length of 100,000 bp in cattle achieves an r^2^ = 0.25, and this block size is approximately three times greater than is found among human populations at the same value of r^2^. Thus, a target marker density for a cattle genotyping array of greater than one marker per 100 kb would position all quantitative trait loci, on average, within 50 kb of a marker for which the expected r^2^ = 0.3 [4]. We consider this density to represent a practical starting point for GWA studies in *Bos taurus*, and at this density, a set of approximately 30,000 variable markers would be sufficient to represent the 3 billion bases of sequence in the bovine genome [6] at an average LD of r^2^≥0.3. Since not all SNP appearing in an assay will be variable in all populations, this target number should be increased to achieve 30,000 variable SNP in each cattle population in which GWA studies are proposed. For comparison, only 80% of the tagSNP on the Infinium® HumanHap550 BeadChip (Illumina, San Diego, CA) are polymorphic in the African Yoruba population [7].

The aim of this study was to develop and characterize a high-density, genome-wide SNP assay for cattle with the power to detect genomic segments harboring inter-individual DNA sequence variation affecting phenotypic traits and for application to GWS, in which an animal's genetic merit is estimated solely from its multilocus genotype [8]. Our specific goals were to develop an assay which would surpass the minimum number of markers required to span the bovine genome at an average marker density of 100 kb, and to achieve an average marker minor allele frequency (MAF) of at least 0.15 among common cattle breeds. Finally, we intended this assay to help validate a set of more than 100 well-characterized SNP markers to facilitate DNA-based approaches to solving common problems such as parentage determination, animal identification, and animal traceability in the event of serious disease outbreak in beef and dairy cattle populations [9], [10]. Eliminating parent misidentification (estimates of up to 25% in cattle; [11] also improves the accuracy of estimation of breeding values [11]–[14]. In a simulation study in which the identity of 11% of the sires was permuted, response to selection for milk production traits was reduced by 11–15% [15]. Thus markers previously identified to have utility for parentage analysis were included in the assay and were validated for the correction of pedigree errors in several breeds of cattle. The performance of the developed bovine SNP assay, now accessible as a commercial genotyping product on a commonly available platform, exceeded these goals.

## Results and Discussion

### SNP Sources

An important goal of this project was to use only public domain SNP and those identified by the authors [16], to avoid intellectual property issues in application of the assay. Table 1 shows the number of SNP from each of the five major sources (Draft, BAC, Bovine HapMap, Interbreed, and RRL; see Methods for a description of the sources) that were available for selection in our design of the Illumina BovineSNP50 assay. The sixth source (Various) included tested, public markers from a variety of published and unpublished experiments. The Draft SNP source (see Methods) had the largest number of putative SNP (approximately 2.1 million), but these lacked MAF information and were unevenly distributed throughout the genome due to the high inbreeding coefficient of the sequenced animal (30%, M.D. MacNeil, pers. comm.). We attempted to validate 101 SNP by targeted resequencing a variety of animals including *L1 Dominette 01449*, the cow used to produce the sequence assembly. Because these 2.1 million SNP were derived from the assembly of sequence data primarily from this animal, she was expected to be heterozygous if the SNP was real. However, we found a low (<50%) conversion rate to polymorphic assays. Through the application of additional quality filters (see Methods) to this SNP source we were able to increase the conversion rate to >85%, but the number of available putative SNP was reduced by nearly 10-fold. Validation studies were also performed to determine SNP conversion rates from the BAC, Interbreed, and RRL sources (Table 1). Genomic positions for all putative SNP were obtained for the design from the draft genome sequence (Btau3.1).

**Table 1:** Summary of performance of SNP by source.

SNP source^1^	Number of SNP available for selection^2^	Estimated conversion rate (%)^3^	Number of SNP selected for the BovineSNP50^4^ (% of assay)	Number of SNP producing calls (%)^5^	Number confirmed SNP (%)^6^	Average MAF^7^
Draft	235,725	85	10,244 (17.6)	9,361 (91.4)	9,284 (99.1)	0.24
Interbreed	73,127	84	6,035 (10.3)	5,493 (91.0)	5,244 (95.5)	0.24
BAC	36,387	82	1,526 (2.6)	1,409 (92.3)	1,239 (87.9)	0.24
RRL	65,180	92	25,833 (44.3)	23,840 (92.3)	21,914 (91.9)	0.25
HapMap	29,853	100^8^	13,236 (22.7)	12,613 (95.3)	12,503 (99.1)	0.26
Parentage	121	100^8^	121 (0.2)	116 (95.9)	116 (100)	0.31
Various	4399	100^8^	1341 (2.3)	1,169 (87.2)	1,083 (92.6)	0.26
**Total**	444,792	NA^9^	58,336	54,001 (92.6)	51,383 (95.1)	0.26

1 Sources of SNP are defined in Materials and Methods.

2 The number of SNP input to the spacing/selection algorithm.

3 Percent of markers detected as polymorphic in validation studies which tested from 48 to 25,125 SNP.

4 BovineSNP50 is the name of the developed high-density genotyping assay.

5 Number of markers that produced genotype calls (>90% call rate) among the 556 tested animals.

6 Number of SNP for which at least one animal was heterozygous among the 556 tested animals and percent of the total number of markers producing genotype calls.

7 Average minor allele frequency among the 556 animals in the validation panel.

8 SNP selected from these sources had previously been shown to be informative in some populations.

9 NA = not applicable.

### Assay Design

Selection of SNP for incorporation into the assay was performed using a novel algorithm applied to SNP that had been assigned to mutually exclusive “waves” defined by a number of features (see Methods and Table S1). The number of beads required to interrogate a SNP (i.e., Infinium I or II class SNP requiring two or one beads, respectively) received heavy influence in defining the waves. From a total of 444,792 SNP, 58,336 were selected to be placed on the BovineSNP50 assay (Table 2, Table S5) to use the 60,800 beadtypes available. The proportion of Infinium II SNP was 87% from all SNP sources, however, the SNP selected for the BovineSNP50 included over 96% Infinium II assays with the remaining 4% being Infinium I (Table S2). The largest number of SNP selected for the BovineSNP50 assay came from the RRL source (Table 1). The primary reason for this result was that the RRL was the largest source of markers with estimated MAF (Table 1). The assay includes 114 of the 121 parentage SNP markers and five SNP associated with two genes previously described to be related to economically important traits: calpastatin (Casas et al. 2006) and bactericidal/permeability-increasing protein (Connor et al. 2008).

**Table 2:** Relationship between Illumina design scores and SNP performance.

Design score	No. SNP submitted	No. SNP passing assay production pipeline (%)	No. high quality SNP called (%)
≥0.9	41,715	40,748 (97.7)	39,390 (96.7)
0.8–0.9	11,855	11,574 (97.6)	10,705 (92.5)
0.7–0.8	3146	3061 (97.3)	2699 (88.2)
0.6–0.7	1216	1170 (96.2)	923 (78.9)
0.5–0.6	382	372 (97.4)	262 (70.4)
ND*	22	22 (100.0)	22 (100.0)
Total	58,336	56,947 (97.6)	54,001 (92.6)

ND*: Design scores were not determined for 5 gene coding SNP and 17 Parentage SNP failed in the design.

**Figure 1:**
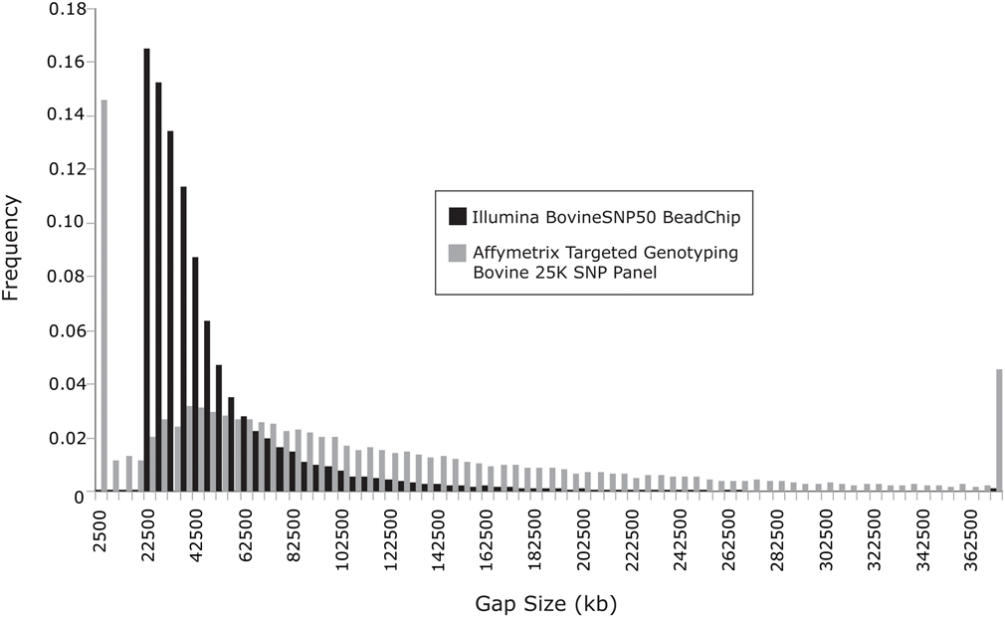
The BovineSNP50 assay has a compact gap distribution ideal for genome wide association studies as compared to the Affymetrix 25 K SNP panel that has an excess of adjacent markers either too close or too far apart, leaving large sections of the genome unrepresented on the assay.

A histogram of the inter-SNP spacing distribution (Figure 1) for the BovineSNP50 assay, as predicted by mapping the SNP onto the Btau3.1 genome assembly, demonstrates that we achieved an even marker distribution across the genome with a median distance of 37 kb between SNP. The histogram also contrasts the inter-SNP distance of the BovineSNP50 assay with that of the Affymetrix GeneChip® Bovine Genome Array [17], which has larger proportions of physically close and distant markers. The contrast between distributions underscores the utility of our method for SNP selection, with its focus on inter-marker distance.

### Performance of the BovineSNP50 assay

Performance metrics for individual SNP and the Illumina platform were tested by genotyping a panel of 576 animals, including those collected for the Bovine HapMap project [17] (Table S3). This panel included 392 animals from 14 taurine dairy and beef breeds, 73 animals from three breeds of predominantly indicine background, 48 animals from two breeds that are taurine×indicine composites, and 45 animals from two African breeds; one a tropically adapted taurine and the other an ancient taurine×indicine composite. For many of the breeds, individuals were sampled from more than one continent to contribute to allele frequency estimation for the global cattle population. Additionally, the panel included 16 additional samples so that Mendelian transmission of alleles (Table S3) and reproducibility could be assessed. This resulted in 48 parent-child relationships, 44 trios where both parents and an offspring were genotyped, and two replicate individuals.

**Figure 2:**
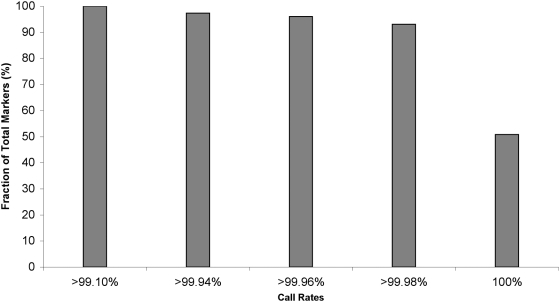
Distribution of SNP by call rates on the BovineSNP50 assay. The overall call rate for all markers exceeded 99.1% and more than 90% had call rates above 99.98%.

The BovineSNP50 assay interrogated 54,001 markers, of which 27,422 markers had a 100% call rate, and less than 3% had call rates below 99.94% (Figure 2) after excluding four poor quality samples. The average call rate for individual samples was greater than 97.5% and 85% of samples had call rates above 98.8%. Fidelity in parent-child transmission was 99.94% concordance, and for trios was 99.95% (Table S4). The replicated samples produced only one discordant genotype among 107,412 genotypes indicating an assay replication success exceeding 99.99%. We conclude that the BovineSNP50 genotyping array provides a robust resource for genome-wide, high-density SNP genotyping of cattle and for population genetic analyses of closely related artiodactyl species.

### Design Scores and Assay Performance

At the design stage, each SNP was processed and assigned an Illumina Infinium design score purported to relate to assay performance. The design score for each SNP on the BovineSNP50 assay was evaluated relative to SNP performance in the genotypes produced for the 576 animal panel (Table S3). We found that the Illumina Infinium® design score accurately predicted assay success (Table 2) despite a few SNP drop outs (<3%) during assay manufacture which were independent of design score. The Infinium I assays (which require two beads to interrogate a SNP) had lower performance than did the Infinium II assays (which require only one bead) (Table 3), regardless of SNP source (Table 4). Since the number of SNP that can be assayed on the iSelect platform is maximized by biasing SNP selection towards Infinium II, this result indicates that SNP conversion to successful assays will also be maximized by this strategy.

**Table 3:** Comparison of overall SNP performance between Infinium I and Infinium II assays.

Design Scores	Number of SNP submitted	SNP passed assay production pipeline		High quality SNP called	
		# SNP	%	# SNP	%
**Infinium I**					
0.9–1.0	1689	1557	92.2	1493	88.4
0.7–0.8	641	597	93.1	551	86.0
0.8–0.9	448	419	93.5	391	87.3
0.6–0.7	131	124	94.7	107	81.7
0.5–0.6	0				
**Infinium II**					
0.9–1.0	40,676	39,826	97.9	38,444	94.5
0.7–0.8	13,810	13,499	97.8	12,377	89.6
0.8–0.9	10,909	10,666	97.8	9884	90.6
0.6–0.7	989	954	96.5	743	75.1
0.5–0.6	339	329	97.1	232	68.4

**Table 4:** Percent success of Infinium I and Infinium II assays derived from the same SNP pools.

Waves Compared^1^	# Infinium II	# Infinium I	% Successful Infinium II	% Successful Infinium I
7 vs 6	1570	188	91.34	82.45
3 vs 9	6464	487	92.95	87.47
11 vs 13	9863	186	91.68	86.02
Total	48,630	2312		

1 See Table S5 for wave definition.

### Characterization of Cattle Breeds using the BovineSNP50 assay

Utility of the SNP set was examined by characterizing SNP allele frequencies among the multi-breed panel, revealing that 51,383 or nearly 95% of the 54,001 called SNP were polymorphic among the 558 cattle (Table S5), with an average minor allele frequency of 0.26 across the entire set. Examining the SNP sources revealed significant differences in the rate of conversion to polymorphic SNP. Four SNP sources were notable in having conversion rates over 98% when characterized across all cattle and outgroup animals: those that had previously been characterized by the Bovine HapMap Consortium [17], those previously screened in smaller genotyping experiments, filtered SNP derived from the assembly of shotgun reads generated from the sequenced cow (*L1 Dominette 01449*), and a set of highly selected, and curated SNP designed to be polymorphic and informative across a broad array of cattle breeds that have been proposed as parentage verification and traceablility SNP [9].

The average MAF when determined across all breeds for the parentage markers was 0.31, considerably higher than the rest of the markers in the assay. The RRL SNP produced a higher conversion rate than did any of the other SNP sources which lacked MAF estimates, although with a substantially lower average MAF than when these markers were validated by genotyping the SNP discovery animals [16]. This primarily reflects the increased diversity of the taurine and indicine cattle that were genotyped here relative to the taurine cattle used in the discovery population. However, these results strongly support our conclusion that the approach of combining RRL with high-throughput sequencing for SNP discovery and MAF estimation is, at present, the most efficient method for generating high quality SNP. Nevertheless, these results also serve as a reminder that the animals selected for SNP discovery should closely represent the populations in which the assay is to be used.

Average MAF was homogeneous among the taurine breeds (0.26±0.01; Table 5), consistent with previous observations that, while individual SNP may show considerable variation in MAF between breeds, on average, SNP are equally informative among breeds. After excluding the Simmental and Gelbvieh breeds in which only three animals were genotyped, the range of informative SNP (78–91%) was higher than the range of average MAF (0.24–0.27) within taurus breeds. While MAF and percent of polymorphic markers remained high in the taurine×indicine composite animals and the average MAF for polymorphic markers was 0.24 in the African breeds, fewer markers were polymorphic in the African breeds. However, both average MAF and the percent of informative markers were significantly reduced in the indicine breeds (Table 5, Figure S1).

**Table 5:** Minor allele frequencies.

Breed	Avg MAF^1^	Informative^2^	Hetero-zygous^3^	MAF				
				0.1	0.2	0.3	0.4	0.5
***Taurine***								
Hereford	0.27	0.89	0.29	0.15	0.17	0.15	0.21	0.21
Charolais	0.26	0.91	0.31	0.15	0.20	0.18	0.20	0.19
Holstein	0.26	0.90	0.31	0.16	0.17	0.18	0.20	0.20
Piedmontese	0.26	0.89	0.31	0.15	0.18	0.19	0.17	0.20
Norwegian Red	0.26	0.88	0.31	0.13	0.20	0.17	0.20	0.18
Limousin	0.25	0.90	0.30	0.17	0.19	0.17	0.19	0.19
Romagnola	0.25	0.84	0.28	0.16	0.18	0.17	0.16	0.18
Angus	0.25	0.89	0.30	0.16	0.18	0.16	0.19	0.19
Red Angus	0.26	0.84	0.30	0.12	0.19	0.15	0.20	0.18
Guernsey	0.25	0.80	0.27	0.13	0.19	0.15	0.17	0.16
Jersey	0.24	0.78	0.26	0.17	0.16	0.14	0.16	0.15
Brown Swiss	0.25	0.80	0.27	0.16	0.16	0.16	0.15	0.16
Simmental	0.3	0.62	0.30	0.00	0.24	0.00	0.25	0.12
Gelbvieh	0.3	0.65	0.30	0.00	0.25	0.00	0.26	0.13
**Taurine×Indicine**								
Beefmaster	0.26	0.92	0.32	0.16	0.19	0.19	0.17	0.20
Santa Gertrudis	0.25	0.91	0.30	0.18	0.19	0.18	0.17	0.19
**African**								
Sheko	0.24	0.75	0.25	0.15	0.17	0.13	0.16	0.14
N'dama	0.24	0.64	0.20	0.14	0.15	0.11	0.13	0.11
***Indicine***								
Brahman	0.18	0.76	0.19	0.28	0.19	0.11	0.10	0.08
Gir	0.19	0.59	0.16	0.20	0.14	0.10	0.07	0.08
Nelore	0.19	0.59	0.15	0.21	0.13	0.09	0.08	0.07

1 Average MAF calculated across all loci including the monomorphic SNP within a given breed,.

2 The fraction of informative SNP with MAF≥0.01.

3 The fraction of heterozygous SNP averaged across all animals within a breed.

**Figure 3:**
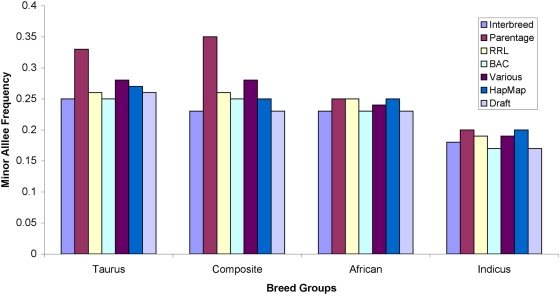
Average MAF by SNP source (see Methods) demonstrates the utility of the assay in taurine, composite, African and indicine cattle.

Plots of MAF by breed type (taurine, indicine, composite and African), and with MAF condensed into 0.1 intervals (Figure 3 and Figure S2), illustrates the utility of designing an assay using markers with known MAF. It can also be seen that all SNP sources utilized in this study were more informative in taurine and composite breed cattle than in indicine and African cattle. A higher proportion of the markers that were polymorphic in the indicine breeds had low MAF. These results demonstrate the utility of the assay for all of the most common beef and dairy breeds in the U.S. and Europe, but indicate that the BovineSNP50 assay will have reduced power for GWA studies within African and indicine breeds. MAF for each SNP in the BovineSNP50 assay within each of the breeds are listed in Table S5. Identifiers in this table include original SNP names and assigned dbSNP identifiers.

### Evaluation of Assay Performance by SNP Source

**Figure 4:**
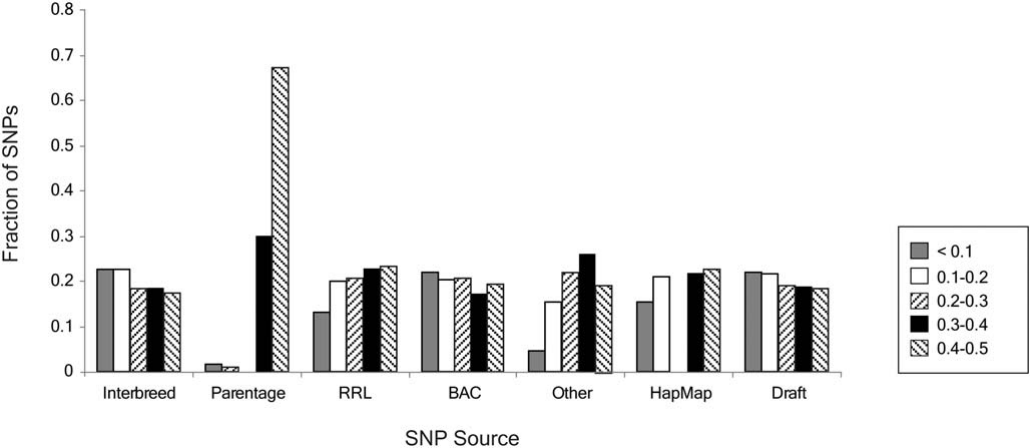
Distribution of SNP minor allele frequency by SNP source.

From the experimental validation of samples of SNP from various sources by sequencing and genotyping (see Methods), we demonstrate that the relative performance of SNP varied according to their origin. By comparing the proportion of informative SNP by group of origin it is clear that the BAC, Draft, and Interbreed SNP had lower MAF than the HapMap and RRL SNP (Figure 4). The predicted and actual performances (rate of polymorphism) of these SNP sources are given in Table 1. The predicted performance of each source of SNP was taken into account when selecting SNP to place on the assay. The SNP were assembled into waves according to whether they were Infinium I or Infinium II, their Illumina Design Score and MAF (Table S1) such that:

A – waves 1, 4, 5 and 6 – High MAF
B – waves 2 and 7 – Low MAF
C – waves 3, 8, 9 and 10 - No MAF available
D – waves 11, 12, 13 and 14 - Draft SNP

**Figure 5:**
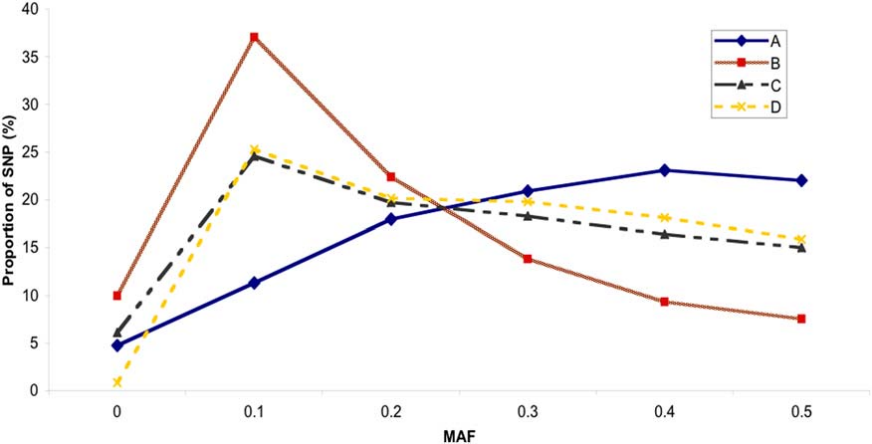
Distribution of SNP MAF by group. A – waves 1, 4, 5 and 6 with High MAF, B – waves 2 and 7 with Low MAF, C – waves 3, 8, 9 and 10 with no MAF available, and D – waves 11, 12, 13 and 14 comprising Draft SNP. (Note: Trend lines drawn only for better illustration).

Figure 5 shows that SNP groups C and D performed very similarly due to the lack of prior MAF estimates. In contrast, SNP groups A and B demonstrate that we were able to identify SNP with low overall MAF which could be de-emphasized in SNP selection for the assay design.

### Parentage SNP

There were 121 parentage SNP available for design, of which, 118 SNP passed the design criteria and 116 were among the 54,001 markers that performed sufficiently reliably for genotyping on the BovineSNP50. Two of the 116 assays were incorrectly targeted to adjacent non-parentage, thus leaving 114 true parentage SNPs on the chip. No genotype inconsistencies were observed among the parent-child pairs and parent-parent-child trios for these markers and all had consistently high call rates. The concordance of genotype calls for the 116 parentage markers genotyped using the BovineSNP50 assay and also obtained from Sanger sequencing reads was evaluated in 8 animals. In 912 possible comparisons, the call rates were 99.6% and 97.1%, respectively, for the BovineSNP50 and Sanger sequencing genotype calls. Discordance between the two assays was identified in 8 of 882 possible comparisons, and thus the error rate was less than 1% (99.1% concordance). The MAF for these markers in taurine breeds ranged from 0.31–0.39. However, these markers had an average MAF of 0.20 within the indicine and African breeds. Plotting MAF within breed type, with MAF condensed into intervals of 0.1 (Figure S3), illustrates the difference in SNP performance between the indicine and remaining breeds. The assay also generated additional candidate SNP for parentage testing that have high MAF across multiple breeds.

### Application to Related Species

The BovineSNP50 assay was applied to a set of DNA samples from six other species within *Bovinae*, including two from different genera, to determine the ancestral allele at each locus. Despite the divergence (1–5 Mya) between these species and *Bos taurus*, over 96% of the SNP produced genotype calls for at least five of the species, including the four species within the genus *Bos* and the Lowland Anoa (*Bubalus depressicornis*) [18]. Only the Cape Buffalo (*Syncerus caffer*), which is estimated to be diverged from *Bos taurus* by 9 Mya, had lower overall genotyping success, but still provided scored genotypes for over 86% of the SNP. Surprisingly, between 1 and 5 percent of the SNP that produced genotype calls were detected as polymorphic in these outgroup species, despite the fact that only 2 or 4 individuals were genotyped within each outgroup species. A small proportion of these variable loci were also common between outgroup species. This result suggests that the origin of some of the SNP chosen for the array predates the divergence of these species. The ancestral allele determined from the analysis of the outgroup samples is provided in Table S5.

### Detection of CNVs in Cattle

The Illumina genotyping software module for detecting copy number variations (CNV) (cnvPartition v1.0.2) was used to analyze the genotyped samples. The resulting CNV were compared to arrayCGH (Nimblegen, Madison, WI) results produced from the DNA samples of eight genotyped animals (G. Liu, pers. comm.). The BeadStudio algorithm performed well for the detection of homozygous deletions which were validated by multiple observations within and across breeds (Table S6). Finally, some of the samples suggested the presence of large insertions and deletions throughout the genome, that indicate a non-uniform signal intensity leading to signal variation being falsely flagged as CNV events. Consequently, high quality CNV commonly segregating in different animals were finally identified after filtering to remove inconsistent samples and retain only those CNVs that identified homozygous deletion events.

We discovered 79 CNV loci from the application of the BovineSNP50 assay to these diverse cattle breeds. Of these, 10 CNV loci overlapped with loci detected from arrayCGH data and these were readily verifiable since the same animals were analyzed using both platforms. The arrayCGH results in turn have been experimentally validated by Q-PCR (unpublished results). However, the two datasets were not completely concordant and had several non-overlapping CNV loci, probably because the arrayCGH experiment has higher resolution due to the availability of more probes (400,000 vs. 54,000) but was performed in fewer breeds. On the other hand, the BovineSNP50 assay was applied to a more diverse set of breeds. More CNV loci were predicted in the African, composite, and indicine breeds than in the taurine breeds, which is consistent with the subspecies divergence and the bioinformatic analyses performed to filter .loci associated with segmental duplications within taurine breeds in the SNP discovery process. However, it is interesting that several of the predicted CNV loci fall within gene boundaries and these loci may play an important role in determining the subspecies divergence. For example, on chromosome 5 (BTA5), CNV loci were detected within olfactory receptor (OR) genes, on BTA12 within two immune response genes UL16 binding protein 2 and ATP-binding cassette, sub-family C (CFTR/MRP), member 4 and also within the two major histocompatibility (MHC) gene regions on BTA23.

### Application to Genome-wide Association Studies

**Figure 6:**
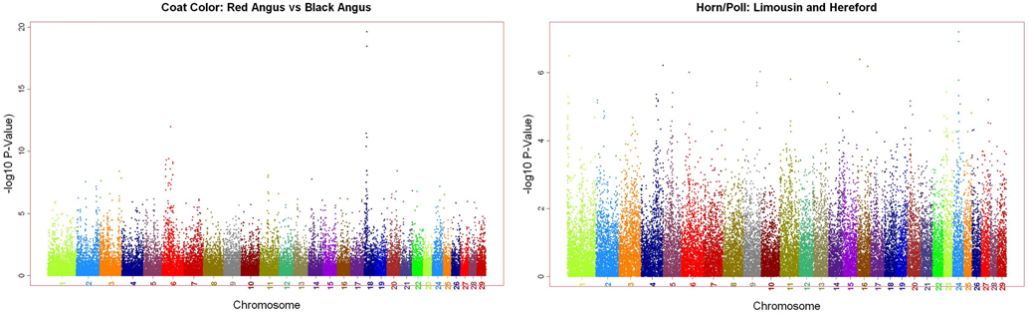
Genome-wide association analyses for (a) coat color based on Fisher's exact test applied to allele frequencies and (b) the POLL locus genotypes based upon a likelihood ratio test for the extent of linkage disequilibrium (r2) between each SNP and the POLL locus.

The BovineSNP50 assay was applied to determine its utility in mapping two Mendelian phenotypes in cattle, black/red coat color and the presence or absence of horns, to identify the chromosomal locations of the loci underlying these traits. The black/red color coat gene *MC1R* [19] is located on BTA18 at position 13,776,888–13,778,639 bp in Btau4.0. To minimize the potential effects of population stratification, we restricted our attention to the Angus breed which is almost exclusively homozygous for the black allele, and Red Angus which is homozygous for the recessive red allele. We performed Fisher's exact test for allele frequency differences between 15 Red Angus and 61 Angus animals using PLINK [20]. The two most significant SNP associations were ARS-BFGL-NGS-31386 at position 13,166,736 bp and ARS-BFGL-NGS-13803 at 13,519,151 bp on BTA18 with p-values of 2.4e-20 and 3.52e-19, respectively (Figure 6A). ARS-BFGL-NGS-31386 is the closest SNP on the BovineSNP50 assay to *MC1R* and no other SNP had a p-value<1e-12. We next analyzed the genotype distribution at ARS-BFGL-NGS-31386 between the two breed groups. The 61 Angus were homozygous *AA* and the Red Angus had 8 *AG* and 7 *GG* animals. Although the black allele is dominant, black-red heterozygote animals often have identifiable red in their coat color, thus the SNP data support the history of strong selection within Angus cattle against red coat color which has driven the black allele to near fixation within the breed. The high frequency of the *AG* heterozygote within Red Angus indicates recombination between the SNP and alleles at the *MC1R* locus, suggesting that the red allele is ancestral.

In cattle, the *POLL* locus is a negative regulator of the horned phenotype. Animals that are homozygous for the ancestral allele are horned while those that carry at least one copy of the derived allele do not have horns and are defined as being polled. Despite considerable research, the mutation responsible for the polled phenotype has yet to be identified. Horns segregate within both the Limousin and Hereford breeds and we utilized phenotypes on genotyped animals, their parents, mates and progeny provided by the North American Limousin Foundation and the American Hereford Association to establish the genotypes at the *POLL* locus for 21 genotyped Limousin and 21 genotyped Hereford animals. Sufficient progeny were produced in matings to horned animals in all cases to differentiate between heterozygous and homozygous polled animals. Among the Limousin, 3 animals were horned, 12 were established as being heterozygotes and 6 as homozygous polled. Among the Herefords, 11 were horned, 8 were heterozygous and 2 were homozygous polled. To avoid breed stratification effects, we independently analyzed each breed by estimating the squared correlation (r^2^) between alleles at each SNP and the *POLL* locus using maximum likelihood. A combined likelihood ratio statistic was constructed for each SNP by summing the log-likelihoods for each breed, and Figure 6B shows the −log_10_(p) values computed for the across-breed likelihood ratio test of H_o_: r^2^ = 0. Figure 6B shows signal in several regions of the genome as might be expected considering the small sample size. Nevertheless, the centromeric region of BTA1, which is known to harbor the *POLL* locus [21], was identified as having one of the strongest signals within the genome.

The BovineSNP50 assay represents a valuable resource for genetic research in *Bovinae* and has desirable properties of whole genome coverage and minimal variation in SNP spacing. All of the markers were obtained from public sources, so application of the assay should not be constrained by intellectual property considerations. The panel and platform provides robust, reproducible and sequence validated genotype calls across a wide range of breeds. The high average MAF suggests that the assay will be useful for implementing GWA and GWS to identify QTL regions and improve production traits in both beef and dairy cattle. By including parentage markers in the assay and validating their utility across several breeds of cattle we can expect to see their widespread use, replacing the current suite of microsatellite markers used for correcting pedigree errors.

## Methods

### Motivation for SNP Discovery

The design and construction of a high quality multiplexed SNP assay requires large numbers of confirmed SNP segregating with high MAF in the target populations. The publicly available SNP data primarily came from the bovine genome sequencing efforts at the Baylor College of Medicine (BCM) and is comprised of two sets: the first set included 114,958 SNP discovered by comparing random shotgun reads from individuals from six diverse breeds to the Hereford genome assembly. These SNP represent only 56,634 unique DNA sequence reads. Slightly more than half (31,681) of these reads contain a single SNP, while the remaining 24,953 reads contain as many as 17 putative SNP, with a putative SNP every 94 bp, on average, for reads containing multiple SNP. The conversion rate for these Interbreed SNP was estimated to be about 80% from the public Bovine HapMap project efforts and from our validations using MassARRAY® (Sequenom, San Diego, CA) assays. The second source of putative SNP included ∼2.1 million markers derived from the observed heterozygous positions within the cattle genome sequence assembly (Draft SNP). Because the sequenced animal had an inbreeding coefficient of approximately 30%, much of the genome was identical by descent leading to large tracts on many chromosomes where few SNP were identified. Further, from an experimental validation of a random sample of 151 SNP derived from this source we observed that the conversion rate was only 41% (See Supplemental Methods S1), and consequently we concluded that these SNP were unusable for assay development. From these observations, it became clear that we lacked sufficient SNP to develop a 50 K SNP assay that would meet our design requirements using the available SNP resources.

To obtain a large number of high-confidence SNP for assay design, additional SNP sources were assembled by:

- Aligning sequence traces (downloaded from the NCBI trace archive) from Holstein BAC (Bacterial artificial chromosome) and BAC-ends to the Hereford genome assembly.
- Applying high stringency filters to the ∼2.1 million Draft SNP to improve their conversion rate.
- Using a novel reduced representation library (RRL) next-generation sequencing strategy to discover large numbers of genome-wide SNP with high MAF [16].

### SNP Discovery from BAC-end and BAC Sequences

We retrieved all sequence traces for the bovine BAC-end and BAC sequences available at the NCBI trace archive as of September 2006 from the RPCI-42 library, which was derived from a juvenile Holstein bull [22]. These traces were primarily comprised of BAC-end sequences (126,800) and some complete BAC shotgun reads (1,091,070) from BAC that had been sequenced as part of the ENCODE project [23] and for high density linkage disequilibrium analysis as part of the Bovine HapMap project [17]. The sequence traces were processed in the following steps for SNP discovery using a variant of the neighborhood quality standard (NQS) method [24]. We:

- Removed very low quality sequences based on Phred scores.
- Masked vector sequences.
- Masked repeat sequences using RepeatMasker (http://www.repeatmasker.org).
- Aligned the sequence reads to the Btau2.0 assembly (ftp://ftp.hgsc.bcm.tmc.edu/pub/data/Btaurus/) after requiring unique mapping with high significance, i.e., a minimum alignment length of 500 bases with no gaps of more than 2 bp.
- Analyzed the aligned sequences for single nucleotide mismatches between the assembly and the sequence reads and, in those positions, the Phred quality score for the varying base had to be at least 30 and the sequence quality of the neighboring 10 bases had to be at least 20.
- Tested for the absence of repeats within the 100 bases flanking each side of the target SNP.

A total of 89,832 unique putative SNP were identified from this pipeline and a sample of 48 SNP were randomly selected and tested by designing primers and sequencing a panel of 96 individuals on an ABI 3730xl sequencer. The conversion rate for these SNP was 82%. All BAC-derived *in silico* SNP were submitted to NCBI dbSNP (ss# 105235583–105324999).

### Filtering of Draft SNP

Based on the analysis of the results from the Draft SNP validation (See Supplemental Methods S1), we implemented a filtering algorithm, which employed the following steps to identify high confidence SNP for assay design:

- Required at least two copies of each allele within the sequence reads.
- No repeats within 100 bp flanking the SNP.
- Minimum coverage of six sequence reads at the SNP locus.
- Removed SNP detected with a coverage of more than 12 reads (to eliminate paralogs, large repeats and gene families).

Application of these filters reduced the total number of available SNP from 2.1 million to 278,429. A random sample from this filtered SNP set was again tested by designing 48 primer sets and sequencing 16 animals on the ABI3730 platform. The conversion rate among these filtered SNP increased to 85%, meeting our requirement for their inclusion in the design of the assay. Using these simple filtering strategies, this SNP source, while greatly reduced in number, was transformed from very low potential to the source with the second highest conversion rate.

### SNP Discovery by Reduced Representation Sequencing

To obtain highly informative SNP with genome-wide coverage, we constructed three reduced representation libraries [24] from pooled DNA samples: the first DNA pool was from 15 cows representing the most popular U.S. dairy breed, Holstein (HOL); the second pool consisted of DNA from 35 Angus bulls (ANG), the most common beef breed in the U.S.; and the third pool included DNA from at least two bulls each from Charolais, Gelbvieh, Hereford, Limousin, Red Angus and Simmental, the six next most common beef breeds (BEEF). To prepare the libraries, we digested these pools with *Hae*III, isolated fragments in the 70–130 bp size range, and sequenced these using an Illumina Genome Analyzer to a depth of about 22-fold coverage across the libraries. From the sequence alignments we detected more than 62,000 SNP [16] and simultaneously estimated their MAF. Since the sequenced tags produced by the Genome Analyzer were relatively short (25 bp), the flanking sequence used for assay design was derived from the genome sequence assembly (ftp://ftp.hgsc.bcm.tmc.edu/pub/data/Btaurus/).

### Parentage SNP

A panel of 121 SNP had previously been identified to accomplish both DNA based traceback and parentage testing in U.S. and Canadian beef and dairy populations (M.P. Heaton et al., unpublished results). These markers were developed by screening >1000 candidate SNP for high MAF, wide genome spacing, and flanking sequence features that were amenable to assay design. Features that were avoided included large blocks of repetitive elements, high-melting temperature (>75°C) stem-loops near the target SNP, high-frequency indel polymorphisms and adjacent SNP near the target SNP. To identify these flanking sequence features and eliminate SNP likely to be problematic for genotyping, the region surrounding the target SNP was sequenced in a diverse beef and dairy cattle breed panel of 192 individuals. These high-quality SNP were designated for inclusion in the assay design for use in parentage validation. Their details are available at http://cgemm.louisville.edu/USDA/index.html.

### Illumina Design Score Pipeline

Sequences flanking putative SNP were evaluated for design using Illumina's Assay Design Tool (http://www.illumina.com/downloads/Illumina_Assay_Design_Tool.pdf). Potential adjacent SNP within 11 bases were identified by their Btau3.1 assembly coordinates and were indicated by IUPAC codes in the sequence submitted for design. We curated potential designs for final submission to manufacturing to maximize the number of SNP present in the design. In addition, SNP submitted to synthesis were screened with an emphasis on design scores. Final SNP selected for the BeadChip design included approximately 4% Infinium I assays with an average design score of 0.92 while the average design score for the Infinium II assays was 0.95.

### SNP Selection for Assay Design

Selection of SNP for incorporation into the assay was performed using a novel algorithm. First, SNP were grouped into a hierarchy based on a number of criteria including the: anticipated conversion rate, number of beads used to interrogate an SNP, design score, and MAF (Table S1). Conversion rate was assigned to SNP sources based upon genotypic data produced either as part of the Bovine HapMap project or from our experimental validations. In some cases, SNP were considered to be reliable even though no allele frequency data were available. This included validated SNP provided by research groups that did not provide MAF data. For these markers, a MAF of 0.20 was assumed when other SNP within the wave had MAF estimates. In the case of the RRL SNP, we considered the MAF for individual SNP estimated during the discovery process to be reliable. The Illumina design scores were utilized in the wave definition under the assumption that they were positively correlated with the likelihood of a specific SNP converting to an assay. The assay type (Infinium I/Infinium II) also received a very strong weight in the wave definition. The first four waves of SNP selection placed over 75% of the total SNP in the assay, yet only about 35% of the total SNP available from these waves were used (Table S2). All of these SNP were Infinium II to maximize the number of SNP that could be incorporated into the design. In total, 15 different SNP waves supplied markers for inclusion in the assay in their order of desirability. Parentage SNP and SNP diagnostic for QTL were mandatory for inclusion in the assay.

### Algorithm for SNP Selection

**Figure 7:**
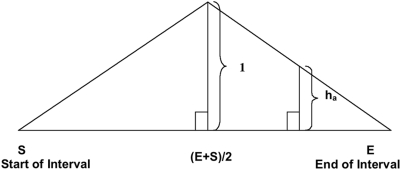
Schema used to produce a weighting factor for assay selection for each candidate SNP depending upon location within a chromosomal interval.

Within each wave of candidate SNP, the objective was to select highly informative SNP that were uniformly distributed throughout the genome. To achieve this goal we used the greedy algorithm approach (http://www.nist.gov/dads/HTML/greedyalgo.html), which sequentially considers the selection of SNP for each genomic interval. While greedy algorithm approaches may not always converge to global optima, they tend to be highly efficient and our empirical data suggest that its application has yielded satisfactory results (Figure 1). In the greedy approach, the each next SNP is iteratively placed into the largest remaining gap on a given chromosome (or unmapped contig) until the target SNP density is achieved or no additional SNP are available to be placed within the remaining segments. In each step, all of the SNP within the interval except those within 20 kb of either end of the interval were considered as candidates (the 20 kb distance was allowed to vary depending on the final spacing results). The SNP with low MAF were penalized relative to SNP with high MAF as their genomic position became increasingly distant from the midpoint of the interval. The scoring function was maximized at the midpoint of the target interval with a value of one and decreased linearly to 0 at each end of the interval (Figure 7). This function can be envisioned as an isosceles triangle with its base spanning the length of the interval, and its height defining the weight factor. The final score for a SNP which determined its eligibility for selection and inclusion within the interval was an adjusted MAF computed as the height of the triangle at the position of the SNP multiplied by the MAF for that SNP. By scaling the interval so that each end is a unit from the midpoint, the triangle in Figure 7 becomes an equilateral triangle, and the height of the triangle, h_a_, at position a ∈[S,E] is:
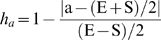
Each SNP was scored based on its MAF, its proximity to the center of the gap in which it would be placed, and the length of the remaining gap:
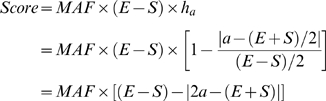
The SNP with the maximum score among all available SNP within a wave was positioned first, and then all scores are recalculated to allow for the gap closure in that round of selection. The process continued until no additional SNP could be added without encroaching upon the minimum distance to the adjacent SNP.

### Association Analysis using PLINK

To determine the ability of the BovineSNP50 assay to map traits, we performed two different analyses that are best suited for the trait segregation patterns. To map the black/red color coat gene, we performed a case-control analysis between the Black and Red Angus breeds using the ASSOC option within PLINK [20]. Fisher's exact test was used to analyze allele frequency differences between 15 Red Angus and 61 Angus animals and the p-values were based on Red Angus to avoid statistical artifacts due to low cell counts. The −log_10_(p) plots were generated using R.

For mapping the horn/poll loci we applied more sophisticated statistical analysis using custom developed software. First we determined genotypes at the horn/poll locus for each of the genotyped animals using mating information and mate/progeny phenotypes and then we performed a pseudo-linkage analysis in which we estimated r^2^ between each SNP and the POLL locus within each breed and finally constructed a statistic that allowed the pooling of breeds without allowing any stratification issues.

We obtained phenotype data for the animals, their parents, all progeny and mates that produced these progeny for the 25 Hereford and 21 Limousin animals that were registered with the American Hereford Association or North American Limousin Foundation, respectively. From this information we were able to infer the *POLL* genotypes of 10 of the 14 polled Hereford and all 18 of the polled Limousin animals. Polled animals that had been produced from a mating of horned and polled parents were obligate carriers, as were animals that produced horned progeny from matings to horned animals. Two Hereford and six Limousin animals had produced no horned progeny in at least 12 matings to horned animals and were classified as homozygous for the polled allele with a level of confidence of at least 99.98%.

We performed case-control association analyses in which SNP allele and genotype frequencies were contrasted between the horned and polled animals within each breed (data not shown) and also a more direct linkage disequilibrium mapping approach in which r^2^ was sequentially estimated between genotypes for each SNP and genotypes at the *POLL* locus by maximum likelihood and the null hypothesis H_o_: r^2^ = 0 versus H_a_: r^2^>0 was tested using a likelihood ratio test. This analysis does not require that the chromosomes be phased to estimate r^2^, since the haplotype frequencies are estimated as one of the parameters within the model [25]. However, rather than employ an E-M approach to maximize the likelihood, we estimated the allele frequencies for the SNP and the *POLL* locus using the usual estimators that are unaffected by the extent of linkage disequilibrium between the loci and then estimated the haplotype frequencies by performing a grid search on the remaining unknown parameter (the remaining three haplotype frequencies are functions of the SNP allele frequencies and the haplotype for which frequency is a parameter in the model). Finally, because the data for the Hereford and Limousin animals defined independent samples, we pooled the likelihood ratio statistics for each tested SNP to produce a whole genome scan for the *POLL* locus in which the SNP and *POLL* locus allele and haplotype frequencies (and thus, also r^2^) were allowed to vary between the breeds, but a single statistic was computed for evidence of LD between the SNP and *POLL* locus across breeds.

## Supporting Information

Table S1 Definitions of waves for SNP used to design the BovineSNP50 assay. <br>
(0.04 MB DOC)

Table S2 Detailed SNP Selection and Performance Results. This table shows the total numbers of SNP available for the assay and the performance of each individual groups of SNPs within the various major cattle sub-species Bos indicus, Bos taurus and outgroup animals
(0.10 MB XLS)

Table S3 Numbers of species, breeds, animals and trios genotyped to characterize the BovineSNP50 assay.
(0.08 MB DOC)

Table S4 Numbers markers producing discordant genotypes with Parent-Child pairs and Parent-Parent-Child trios.
(0.04 MB DOC)

Table S5 The derived ancestral allele along with minor allele frequencies of individual markers within each of the cattle breeds and outgroups.
(19.24 MB XLS)

Table S6 Locations and nature of copy number variation loci detected with the BovineSNP50 assay.
(0.06 MB XLS)

Figure S1 Comparison of minor allele frequency by SNP source for Holstein (blue) and Brahman (red). Panel A) RRL SNNP, B) HapMap SNP, C) BAC-derived SNP, and D) Draft assembly derived SNP.
(0.04 MB DOC)

Figure S2 Distribution of BovineSNP50 SNP by MAF for taurine, indicine, composite and African breeds of cattle by SNP source. Panels A–C) SNP sources (see Methods) with prior MAF estimates, panels D–F) SNP sources without prior MAF estimates.
(0.08 MB DOC)

Figure S3 The minor allele frequency distribution for parentage SNP within representative classes of cattle composite (Beefmaster), beef (Angus), dairy (Holstein) and indicine (Gir) shows that the markers are highly informative among the common taurine beef and dairy breeds and in composites but are least informative within the indicine breed.
(0.02 MB DOC)

Methods S1 Supplementary Methods
(0.03 MB DOC)
