# Supplementary material for: Development and Characterization of a High Density SNP Genotyping Assay for Cattle
Source: PLoS One. 2009 Apr 24;4(4):e5350. doi: 10.1371/journal.pone.0005350 (PMC2669730; doi:10.1371/journal.pone.0005350)
Supplement: Table S1 — Definitions of waves for SNP used to design the BovineSNP50 assay. (0.04 MB DOC) [file pone.0005350.s001.doc]

**Table S1. Definitions of waves for SNP used to design the BovineSNP50 assay.**

| **Wave^1^** | **Assay Type** | **Minimum Design Score** | **Minimum MAF** | **SNP Source** |
| --- | --- | --- | --- | --- |
| 1 | Infinium II | 0.8 | 0.15 | HapMap, RRL, IFASA, USMARC & DPI, INRA |
| 2 | Infinium II | 0.7 | 0.1 | HapMap, RRL, IFASA, USMARC & DPI, INRA |
| 3 | Infinium II | 0.8 | 0 | InterBreed, BAC |
| 4 | Infinium II | 0.5 | 0.2 | HapMap, RRL, IFASA, USMARC & DPI, INRA |
| 5 | Infinium I | 0.8 | 0.15 | HapMap, RRL, IFASA, USMARC & DPI, INRA |
| 6 | Infinium I | 0.6 | 0.2 | HapMap, RRL, IFASA, USMARC & DPI, INRA |
| 7 | Infinium II | 0.6 | 0.05 | HapMap, RRL, IFASA, USMARC & DPI, INRA |
| 8 | Infinium II | 0.6 | 0 | InterBreed, BAC |
| 9 | Infinium I | 0.8 | 0 | InterBreed, BAC |
| 10 | Infinium I | 0.6 | 0 | InterBreed, BAC |
| 11 | Infinium II | 0.8 | 0 | Draft |
| 12 | Infinium II | 0.6 | 0 | Draft |
| 13 | Infinium I | 0.8 | 0 | Draft |
| 14 | Infinium I | 0.6 | 0 | Draft |
| 15 | Infinium I and II | 0 | 0 | Parentage and Genes |

^1^Wave is defined as a pool of SNP formed from different sources according to desirability for incorporation into the design of the BovineSNP50 assay. Waves were processed sequentially to produce the design.
