# Supplementary material for: Development and Characterization of a High Density SNP Genotyping Assay for Cattle
Source: PLoS One. 2009 Apr 24;4(4):e5350. doi: 10.1371/journal.pone.0005350 (PMC2669730; doi:10.1371/journal.pone.0005350)
Supplement: Table S3 — Numbers of species, breeds, animals and trios genotyped to characterize the BovineSNP50 assay. (0.08 MB DOC) [file pone.0005350.s003.doc]

**Table S3**. Numbers of species, breeds, animals and trios genotyped to characterize the BovineSNP50 assay.

| **Breed or species** | **Acronym** | **# Animals** | **# Males** | **# Females** | **# Trios** |
| --- | --- | --- | --- | --- | --- |
| ***Taurine*** |  |  |  |  |  |
| Holstein | HOL | 64 | 52 | 12 | 5 |
| Angus | ANG | 62 | 58 | 4 | 3 |
| Limousin | LMS | 45 | 41 | 4 | 2 |
| Hereford | HFD | 32 | 27 | 5 | 3 |
| Jersey | JER | 28 | 25 | 3 | 3 |
| Charolais | CHL | 26 | 20 | 6 | 3 |
| Brown Swiss | BSW | 24 | 21 | 3 | 3 |
| Piedmontese | PMT | 24 | 16 | 8 | 3 |
| Ramagnola | RMG | 24 | 21 | 3 | 3 |
| Guernsey | GNS | 21 | 1 | 20 | 0 |
| Norwegian Red | NRC | 21 | 16 | 5 | 1 |
| Red Angus | RGU | 15 | 14 | 1 | 1 |
| Gelbvieh | GBV | 3 | 3 | 0 | 0 |
| Simmental | SIM | 3 | 3 | 0 | 0 |
|  |  |  |  |  |  |
| ***Indicine*** |  |  |  |  |  |
| Brahman | BRM | 25 | 12 | 13 | 3 |
| Gir | GIR | 24 | 20 | 4 | 3 |
| Nelore | NEL | 24 | 9 | 15 | 1 |
|  |  |  |  |  |  |
| ***Taurine×Indicine*** |  |  |  |  |  |
| Beefmaster | BMA | 24 | 23 | 1 | 0 |
| Santa Gertrudis | SGT | 24 | 21 | 3 | 3 |
|  |  |  |  |  |  |
| ***African Breeds*** |  |  |  |  |  |
| N'Dama (Taurine) | NDA | 25 | 5 | 20 | 2 |
| Sheko (Ancient Taurine×Indicine hybrid) | SHK | 20 | 6 | 14 | 1 |
|  |  |  |  |  |  |
| ***Outgroup Species*** |  |  |  |  |  |
| Gaur (*Bos gaurus*) | OGR | 4 | 0 | 4 | 0 |
| North American Bison (*Bison bison*) | OBB | 4 | 2 | 2 | 1 |
| Lowland Anoa (*Bubalus depressicornis*) | OWB | 4 | 3 | 1 | 0 |
| Banteng (*Bos javanicus*) | OBJ | 2 | 2 | 0 | 0 |
| Yak (*Bos grunniens*) | OYK | 2 | 2 | 0 | 0 |
| Cape Buffalo (*Syncerus caffer*) | OCB | 2 | ? | ? | 0 |
|  |  |  |  |  |  |
| **Total** |  | 576 | 423 | 151 | 44 |
