# Supplementary material for: Development and Characterization of a High Density SNP Genotyping Assay for Cattle
Source: PLoS One. 2009 Apr 24;4(4):e5350. doi: 10.1371/journal.pone.0005350 (PMC2669730; doi:10.1371/journal.pone.0005350)
Supplement: Table S4 — Numbers markers producing discordant genotypes with Parent-Child pairs and Parent-Parent-Child trios. (0.04 MB DOC) [file pone.0005350.s004.doc]

**Table S4. Numbers markers producing discordant genotypes with Parent-Child pairs and Parent-Parent-Child trios**.

|  | Parent-Child Pairs | |  | Parent-Child-Child trios | |
| --- | --- | --- | --- | --- | --- |
| # Errors | # SNP | % SNP |  | # SNP | % SNP |
| 0 | 51,052 | 99.35 |  | 51,129 | 99.50 |
| 1 | 200 | 0.39 |  | 145 | 0.28 |
| 2 | 34 | 0.07 |  | 18 | 0.04 |
| 3 | 11 | 0.02 |  | 12 | 0.02 |
| 4 | 4 | 0.01 |  | 2 | 0.00 |
| 5 | 2 | 0.00 |  | 5 | 0.01 |
| 6 | 5 | 0.01 |  | 8 | 0.02 |
| 7 | 3 | 0.01 |  | 6 | 0.01 |
| 8 | 2 | 0.00 |  | 9 | 0.02 |
| 9 | 5 | 0.01 |  | 7 | 0.01 |
| 10 | 4 | 0.01 |  | 7 | 0.01 |
| >10 | 64 | 0.12 |  | 38 | 0.07 |
| Total | 51,386 |  |  | 51,386 |  |
