# Supplementary material for: Development and Characterization of a High Density SNP Genotyping Assay for Cattle
Source: PLoS One. 2009 Apr 24;4(4):e5350. doi: 10.1371/journal.pone.0005350 (PMC2669730; doi:10.1371/journal.pone.0005350)
Supplement: Figure S1 — Comparison of minor allele frequency by SNP source for Holstein (blue) and Brahman (red). Panel A) RRL SNNP, B) HapMap SNP, C) BAC-derived SNP, and D) Draft assembly derived SNP. (0.04 MB DOC) [file pone.0005350.s007.doc]

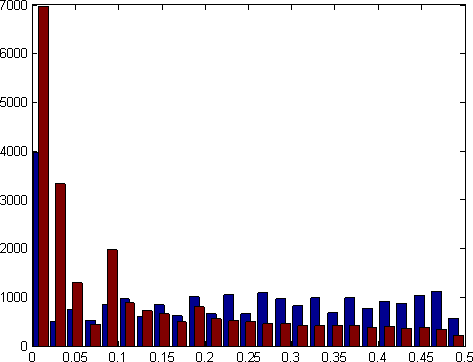

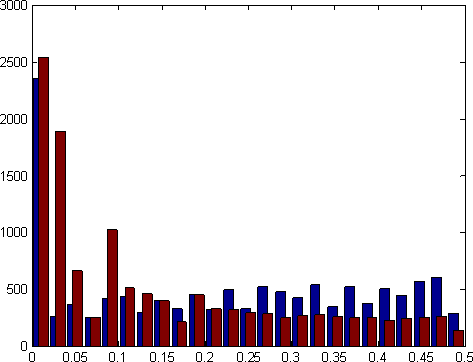

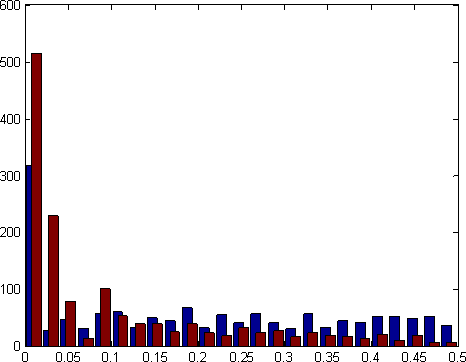

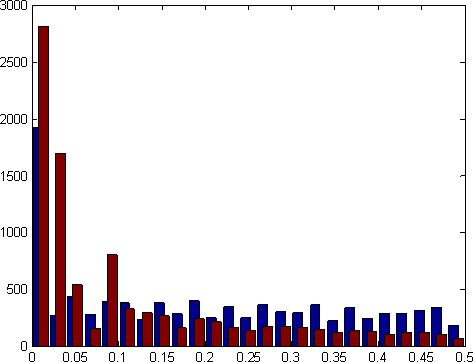


**Figure S1.** Comparison of minor allele frequency by SNP source for Holstein (blue) and Brahman (red). Panel A) RRL SNNP, B) HapMap SNP, C) BAC-derived SNP, and D) Draft assembly derived SNP.

A

B

C

D
