# Supplementary material for: Development and Characterization of a High Density SNP Genotyping Assay for Cattle
Source: PLoS One. 2009 Apr 24;4(4):e5350. doi: 10.1371/journal.pone.0005350 (PMC2669730; doi:10.1371/journal.pone.0005350)
Supplement: Figure S2 — Distribution of BovineSNP50 SNP by MAF for taurine, indicine, composite and African breeds of cattle by SNP source. Panels A–C) SNP sources (see Methods) with prior MAF estimates, panels D–F) SNP sources without prior MAF estimates. (0.08 MB DOC) [file pone.0005350.s008.doc]

A

B

C

D

E

F

**Figure S2.** Distribution of BovineSNP50 SNP by MAF for taurine, indicine, composite and African breeds of cattle by SNP source. Panels A-C) SNP sources (see Methods) with prior MAF estimates, panels D-F) SNP sources without prior MAF estimates.
