# Supplementary material for: Development and Characterization of a High Density SNP Genotyping Assay for Cattle
Source: PLoS One. 2009 Apr 24;4(4):e5350. doi: 10.1371/journal.pone.0005350 (PMC2669730; doi:10.1371/journal.pone.0005350)
Supplement: Figure S3 — The minor allele frequency distribution for parentage SNP within representative classes of cattle composite (Beefmaster), beef (Angus), dairy (Holstein) and indicine (Gir) shows that the markers are highly informative among the common taurine beef and dairy breeds and in composites but are least informative within the indicine breed. (0.02 MB DOC) [file pone.0005350.s009.doc]

**Figure S3.** The minor allele frequency distribution for parentage SNP within representative classes of cattle composite (Beefmaster), beef (Angus), dairy (Holstein) and indicine (Gir) shows that the markers are highly informative among the common taurine beef and dairy breeds and in composites but are least informative within the indicine breed.
