# Supplementary material for: Development and Characterization of a High Density SNP Genotyping Assay for Cattle
Source: PLoS One. 2009 Apr 24;4(4):e5350. doi: 10.1371/journal.pone.0005350 (PMC2669730; doi:10.1371/journal.pone.0005350)
Supplement: Methods S1 — Supplementary Methods (0.03 MB DOC) [file pone.0005350.s010.doc]

**Supplementary Methods**

**Draft SNP Validation:**

We designed 151 MassARRAY® (Sequenom, San Diego, CA) assays, which were attempted on 16 animals to estimate the conversion rate of the 2.1 million Draft SNP markers. These SNP were derived from the observed heterozygous positions in the sequence assembly (Btau2.0 build) of a single Hereford cow *L1 Dominette 01449* and from BAC sequences from her sire. Hence we included DNA from the Dominette cow among the genotyped animals. In this test, only 47 SNP (31%) were identified as being heterozygous in Dominette as expected and also produced genotype calls in the other animals. The rest of the assays failed for multiple reasons: (i) 37 assays (24%) failed to produce genotypes for any of the animals, (ii) four assays (3%) produced heterozygous calls for all animals, (iii) 13 (11%) prodcued no valid genotype calls in animals other than Dominette, (iv) 40 assays (26%) were all homozygous, and (v) unexpectedly, 10 assays failed to demonstrate heterozygosity in Dominette as anticipated but produced heterozygous calls in some of the other animals. These assays may reflect SNP that were discovered due to genotype differences between Domino and his daughter, Dominette.

To determine the influence of repeat elements in assay failures we partitioned the 151 assays into three groups:

1. Sequences with no repetitive elements (N= 43)
2. Sequences containing repetitive elements but not at the SNP locus (N=46)
3. SNP locus located within a repeat sequence (N= 62)

Among SNP located within repeat sequences (Set 3) the conversion rate was only 30%. For the SNP in Sets 1 and 2 the expected heterozygosity of Dominette was observed in only 38% of cases. However, the conversion rate among these SNP was increased by requiring at least two sequences to contain the appropriated base pair for each of the SNP alleles among all of the sequence reads used to produce the assembly.
